# Energetic benefits of enhanced summer roosting habitat for little brown bats (*Myotis lucifugus*) recovering from white-nose syndrome

**DOI:** 10.1093/conphys/cov070

**Published:** 2016-02-26

**Authors:** Alana Wilcox, Craig K. R. Willis

**Affiliations:** Department of Biology and Centre for Forest Interdisciplinary Research, University of Winnipeg, Winnipeg, MB, Canada

**Keywords:** Bat house, habitat enhancement, habitat modification, *Pseudogymnoascus destructans*

## Abstract

Habitat modification can improve outcomes for imperilled wildlife. Insectivorous bats in North America face a range of conservation threats, including habitat loss and white-nose syndrome (WNS). Even healthy bats face energetic constraints during spring, but enhancement of roosting habitat could reduce energetic costs, increase survival and enhance recovery from WNS. We tested the potential of artificial heating of bat roosts as a management tool for threatened bat populations. We predicted that: (i) after hibernation, captive bats would be more likely to select a roost maintained at a temperature near their thermoneutral zone; (ii) bats recovering from WNS at the end of hibernation would show a stronger preference for heated roosts compared with healthy bats; and (iii) heated roosts would result in biologically significant energy savings. We housed two groups of bats (WNS-positive and control) in separate flight cages following hibernation. Over 7.5 weeks, we quantified the presence of individuals in heated vs. unheated bat houses within each cage. We then used a series of bioenergetic models to quantify thermoregulatory costs in each type of roost under a number of scenarios. Bats preferentially selected heated bat houses, but WNS-affected bats were much more likely to use the heated bat house compared with control animals. Our model predicted energy savings of up to 81.2% for bats in artificially heated roosts if roost temperature was allowed to cool at night to facilitate short bouts of torpor. Our results are consistent with research highlighting the importance of roost microclimate and suggest that protection and enhancement of high-quality, natural roosting environments should be a priority response to a range of threats, including WNS. Our findings also suggest the potential of artificially heated bat houses to help populations recover from WNS, but more work is needed before these might be implemented on a large scale.

## Abstract (precis)

Enhancement of summer habitat could improve survival and reproduction by bats recovering from white-nose syndrome (WNS). We found that captive little brown bats (Myotis lucifugus) recovering from WNS preferentially selected heated bat houses and we calculated a dramatic reduction in energy costs for bats in heated roosts.

## Introduction

Habitat quality influences survival, reproduction and fitness of wildlife (Nager and Noordwijk, 1992; Bryan and Bryant, 1999), and protection of high-quality habitat is important for conservation and recovery of threatened species or populations (Knaepkens *et al.*, 2004; Savard and Robert, 2007; Kapust *et al.*, 2012). Beyond protection of existing habitat, habitat enhancement involves modifying the local environment to improve conditions for conservation of individual species or overall biodiversity (Weller, 1989). Habitat enhancement can be used to target many aspects of the biology of threatened species, including nesting or egg laying (Knaepkens *et al.*, 2004; Savard and Robert, 2007; Kapust *et al.*, 2012), growth rate and body size/condition (Riley and Fausch, 1995), and competition with invasive species (Grarock *et al.*, 2013, 2014). Ultimately, these effects can lead to increased survival, enhanced population growth rates and improved population stability and recovery (Riley and Fausch, 1995).

For endothermic species, enhancing habitat to reduce thermoregulatory costs can improve survival and fitness and has potential as a management tool for threatened populations (e.g. Schifferli, 1973; Nager and Noordwijk, 1992; Yom-Tov and Wright, 1993; Bryan and Bryant, 1999; Boyles and Willis, 2010). Warm microclimates can be especially important for successful gestation and improved growth of offspring (Tuttle, 1976; McCarty and Winkler, 1999). Yom-Tov and Wright (1993) found that when nest boxes were heated to 6°C above ambient temperature (*T*_a_) Eurasian blue tits (*Cyanistes caeruleus*) had an energy saving of ∼3.21 kJ over 7 h and interruption in egg laying was reduced. Likewise, in great tits (*Parus major*) a 3.4°C increase above *T*_a_ for 11 h saved birds about 7 kJ, increased the time allocated to incubation, and allowed birds to lay larger eggs (Nager and Noordwijk, 1992; Bryan and Bryant, 1999).

Insectivorous bats are one group of endotherms facing a range of conservation threats, including habitat loss (Kunz and Lumsden, 2003), climate change (Humphries *et al.*, 2002) and disease (Frick *et al.*, 2010; Langwig *et al.*, 2012; Warnecke *et al.*, 2012), and they are also a candidate taxon for a habitat enhancement approach to management. Bats spend most of their lives roosting, and roost microclimate is thought to play a key role in survival and reproductive success (e.g. Fenton and Barclay, 1980; Kunz and Lumsden, 2003; Lausen and Barclay, 2003). In the temperate zone, roost temperature (*T*_roost_) and *T*_a_ will often fall below thermoneutrality (Willis and Brigham, 2005), and many bat species use torpor [i.e. reduced body temperature (*T*_b_) and metabolism] to reduce energetic costs (e.g. Willis *et al.*, 2006). However, torpor can delay parturition and inhibit lactation in female bats (Lewis, 1993; Wilde *et al*., 1995) and is known to inhibit spermatogenesis in male ground squirrels (Barnes *et al.*, 1986). Therefore, although bats employ some torpor during the reproductive season to save energy and, potentially, delay parturition during inclement weather (Willis *et al.*, 2006), they may also select roosts that help them to reduce reliance on torpor (Chruszcz and Barclay, 2002; Lausen and Barclay, 2003). The dependence of bats on warm *T*_roost_ suggests that habitat protection or enhancement targeting roost microclimates could benefit survival, reproduction and population growth for temperate bats.

Roost microclimate could also play a role in population recovery from disease. White-nose syndrome (WNS) is an infectious disease of hibernating bats, caused by the fungus *Pseudogymnoascus destructans* (Lorch *et al.*, 2011; Warnecke *et al.*, 2012) and responsible for unprecedented declines of bats across eastern North America (US Fish and Wildlife Service, 2012). The mechanisms underlying mortality are still not fully understood, but affected bats are emaciated as a result of an increase in arousal frequency and energy expenditure during hibernation (reviewed by Willis, 2015). Some bats survive WNS (Dobony *et al.*, 2011; Maslo *et al.*, 2015) but, unfortunately, survivors exhibit a rapid reversal of immune suppression in spring that appears to cause immune reconstitution inflammatory syndrome, which is characterized by a dramatic inflammatory response, deterioration in physiological condition and, possibly, mortality (Meteyer *et al.*, 2012). Healing from WNS-associated wing damage, in addition to the immune reconstitution inflammatory syndrome response itself, is likely to be energetically expensive (Fuller *et al.*, 2011). Thus, availability of warm roost microclimates could help survivors make it through potentially harsh spring conditions and initiate reproduction earlier in summer, improving survival of their offspring. If survivors exhibit heritable traits that help them to survive the winter with WNS (Willis and Wilcox, 2014; Maslo and Fefferman, 2015), this approach could facilitate evolution of WNS-survival traits in threatened populations (Maslo *et al.*, 2015).

We used a combination of behavioural experiments with a captive colony of little brown bats (*Myotis lucifugus*) and a series of bioenergetic models to test the hypothesis that enhancement of the microclimate of summer roosts could aid management of bat populations imperilled by WNS and other threats. We predicted that: (i) during the post-hibernation period, captive bats would be more likely to select sites with *T*_roost_ maintained near their thermoneutral zone compared with colder roosts; (ii) bats recovering from WNS at the end of hibernation would show a stronger preference for a heated roost compared with healthy individuals; and (iii) heated roosts would result in biologically significant energy savings for bats compared with natural *T*_roost_.

## Materials and methods

### Roost selection experiment

All procedures were conducted under Manitoba Conservation and Ontario Ministry of Natural Resources permits and approved by the University of Winnipeg Animal Care Committee. Between 28 April and 5 May 2013, 32 adult male little brown bats (hereafter, ‘infected’) were captured from two WNS-positive mines <90 km apart near Renfrew, Ontario (45.47°N, 76.68°W) and Gatineau, Québec (45.48°N, 75.65°W), Canada. These bats were used in several studies examining the recovery phase from WNS and, of these individuals, 11 were available for use in the present study. At the time of capture, bats were weighed (CS200 portable scale; Ohaus Corporation, Pine Brook, NJ, USA, accuracy ±0.1 g), and their forearm length was measured using digital callipers (digital caliper; Mastercraft, Vonore, TN, USA). All bats were outfitted with temperature dataloggers (DS1922L-F5 Thermochron iButton; Maxim, San Jose, CA, USA) modified to reduce mass following Lovegrove (2009) and Reeder *et al.* (2012). Temperature dataloggers were attached in the interscapular region using a latex-based adhesive (Osto-Bond; Montreal Ostomy Centre, Vaudreuil-Dorion, QC, Canada), and a unique symbol was marked on each datalogger for easy identification of individuals. The dataloggers were used for another study to record *T*_b_ during late winter and early spring, and datalogger memories were full by the time our behavioural experiment began. However, we did not remove them from bats before our experiment in order to reduce handling stress. Thus, no *T*_b_ data are reported in this paper. Bats were held in cloth bags inside hepa-filtered animal carriers stored in a temperature-controlled cabinet for transport to the University of Winnipeg. Bats were later banded with a uniquely numbered, lipped aluminum forearm band (Porzana Ltd, Icklesham, East Sussex, UK).

On 28 May 2013, 39 male little brown bats (hereafter, ‘control’) were captured from a WNS-negative cave in the northern-interlake region of Manitoba, Canada (53.20°N, 99.30°W). Of these individuals, 26 were available for this study. Bats were tagged and placed in cloth bags suspended in a plug-in ventilated cooler for transport to the University of Winnipeg. Morphometric measurements were obtained as described for infected bats. Bats from both the infected and control groups were housed over the summer until the beginning of hibernation on 11 October 2013. We confirmed the infection status of all individuals from both infected (all individuals were positive for *P. destructans*) and control (all individuals were negative) groups via quantitative PCR conducted at the Center for Microbial Genetics and Genomics at Northern Arizona University (Lorch *et al.*, 2010).

The holding room was maintained at 18°C and 60% relative humidity, and lights were set for a natural spring photoperiod (light:dark = 11:13 h) with a graduated transition from lights on to lights off. Infected and control bats were housed in separate but identical flight cages 2.24 m^2^ long × 1.01 m wide × 2.42 m high. Separate cages eliminated the possibility of infection of control bats because *P. destructans* does not spread without physical contact in the laboratory (Lorch *et al*., 2010). Each flight cage was equipped with two custom-built single-chambered bat houses (44 cm × 6.3 cm × 60 cm). The back of one bat house in each cage was lined with an electrical heating coil (Exoterra Temperature Heating Cable, 12 V; Rolf C. Hagen Group, Mansfield, MA, USA) controlled using an electronic temperature controller set to 30°C (Ranco Nema 4× Electircal Temperature Control; Invensys, Plain City, OH, USA), slightly below the lower critical temperature for little brown bats (i.e. 32°C; Stones and Wiebers, 1965; Speakman and Thomas, 2003). In the infected flight cage, the heated bat house was mounted on 7 May 2013, whereas the unheated bat house was provided on 21 May 2013 because it was not yet available when infected bats were collected. In the flight cage housing control bats, both the heated and unheated bat houses were installed on 21 May 2013. Throughout captivity, bats were provided with mealworms (*Tenebrio molitor*) and water *ad libitum* on tables in the centre of each flight cage. Bats were collected from inside bat houses for weighing about every 2 days. Between 9 June and 31 July 2013, while collecting bats for these weighing sessions, we recorded the number of bats in each type of bat house in each flight cage.

Although they are among the species most affected by WNS and, therefore, important to study, little brown bats are difficult to maintain in captivity during the active season (Lollar, 2010). Between 3 May 2013 and 28 July 2013, some infected (*n* = 2) and control bats (*n* = 17) exhibited abnormal behaviour, including impaired and weakened gait, inability to fly, lack of feeding, and a decline in body mass. Bats exhibiting these symptoms were isolated in smaller nylon mesh cages (20.3 cm × 20.9 cm × 24.1 cm) to improve access to food and water and facilitate monitoring. If their condition continued to decline, as indicated by a loss of >5% body mass, they were anaesthetized using isoflurane in oxygen (5%) and euthanized via CO_2_ asphyxiation. Pathology showed no bacterial or viral infection, and bats did not exhibit signs of fatty liver syndrome, which can cause the behavioural patterns we observed in captive bats as a combined result of excess food consumption and stress (Lollar, 2010; Olsson and Barnard, 2009). In addition to this unidentified problem, between 23 July and 31 July 2013 two bats were isolated for lower abdominal swelling that turned out to be a bacterial infection caused by *Proteus morganii*. Therefore, all bats were given an oral administration of enrofloxacin (0.1 mL day^−1^) and cefazolin (0.1 mL day^−1^). These challenges led to fluctuating numbers of bats in each cage as individuals were moved between the flight cage and isolation, and a gradual decline in numbers in each flight cage as bats were removed from the study (*n* = 8 bats per group by the end of the study). As a result of these fluctuating numbers, we quantified roosting preferences as the proportion of bats in each flight cage found roosting in the heated vs. unheated houses during the times when we captured bats for weighing.

### Energetic models

We used bioenergetic models to quantify potential energy savings that could be provided by artificially heated bat houses based on *T*_a_ measurements from central Manitoba, Canada, during post-hibernation. Pregnant female bats select maternity roosts with *T*_a_ values that help to reduce energy expenditure, increase time spent in normothermia and avoid, but not eliminate, expression of torpor (e.g. Barclay, 1982; Dzal and Brigham, 2013). Female bats appear to select roosts that facilitate torpor in the early morning when *T*_roost_ is lowest and then gradually rewarm throughout the day, often reaching values of *T*_roost_ well above outside *T*_a_, to help maintain normothermia (Dzal and Brigham, 2013).

We calculated all predicted metabolic rates (MRs) as mass-specific oxygen consumption (mass-specific ${\dot{V}}_{O_{2}}$; in millilitres of  O_2_ per gram per hour). We calculated normothermic energy expenditure (*E*_norm_) using the following equation:

(1) $$E_{\mathrm{norm}}=\mathrm{BMR}+(T_{\mathrm{lc}}-T_{a})C_{\mathrm{norm}}$$

where BMR is the basal metabolic rate (Willis *et al*., 2005), *T*_lc_ is the lower critical temperature of the thermoneutral zone, *T*_a_ is ambient temperature, and *C*_norm_ is thermal conductance at normothermia (Humphries *et al.*, 2002, 2005). We assumed that the decline in *T*_b_ from *T*_norm_ to *T*_tor_ during entry into torpor cost 67.2% of the cost of warming, following Equation 4 of Thomas *et al*. (1990). During torpor, MR and *T*_b_ decline to a minimal set-point temperature (*T*_tor-min_), at which metabolic heat production is required to defend torpid *T*_b_ (Humphries *et al.*, 2002; Geiser, 2004, 2013). Therefore, we followed Humphries *et al*. (2002) and used two equations to quantify predicted energy expenditure during torpor depending on whether *T*_a_ was lower or higher than *T*_tor-min_, as follows

(2) $$\mathrm{When}\;\;\;T_{a}\;\;>\;\;T_{\mathrm{tor}-\min},\;\;E_{\mathrm{tor}}=\mathrm{TM}R_{\min}{Q_{10}}^{(T_{a}-T_{\mathrm{tor}-\min})/10}$$

(3) $$\mathrm{When}\;\;\;T_{a}\leq T_{\mathrm{tor}-\min},\;\;E_{\mathrm{tor}}=\mathrm{TM}R_{\min}+(T_{\mathrm{tor}-\min}-T_{a})C_{t}$$

where *Q*_10_ is the change in torpid metabolic rate (TMR) over a 10°C change in *T*_a_, and *C*_t_ is thermal conductance below *T*_tor-min_ (Humphries *et al.*, 2002, 2005). We calculated metabolic costs of active arousals from torpor as the energy required to increase *T*_b_ from *T*_tor_ to *T*_norm_ based on the specific heat capacity of tissues (*S*; 0.131 ml O_2_ g^−1^ °C^−1^) and mass, following the equation from McKechnie and Wolf (2004):

(4) $$E_{\mathrm{ar}}=S(T_{\mathrm{norm}}-T_{\mathrm{tor}-\min})M_{b}+D_{\mathrm{rewarm}}\left(\frac{\mathrm{TMR}+(\mathrm{BMR}-\mathrm{TMR})}{2}\right)$$

Note that the second term in Equation 4 quantifies the rate of rewarming as a linear function with *D*_rewarm_ as the duration of rewarming (McKechnie and Wolf, 2004). To estimate TMR for Equation 4, we used the equation from McKechnie and Wolf (2004):

(5) $$\mathrm{TMR}=\mathrm{TM}R_{\min}+C_{\mathrm{tor}}(T_{\mathrm{tor}-\min}-T_{a})$$

The metabolic cost of a passive arousal (i.e. when *T*_a_ or solar radiation would have warmed a roost) was calculated as 1.04 × BMR (Willis *et al.*, 2004).

To convert values of mass-specific ${\dot{V}}_{O_{2}}$ into SI units of heat production or energy expenditure (joules per gram per hour), we used Equation 3 from Campbell *et al*. (2000), which accounts for differences in the catabolism of lipids (L), carbohydrates (C) and proteins (P) in the diet, as follows:

(6) $$\begin{array}{rl} \&\mathrm{Heatproduction} & =(17.71P+20.93C+19.55L)\\ & \times \mathrm{mass}-\mathrm{specific}\;{\dot{V}}_{O_{2}} \end{array}$$

Little brown bats are generalist insectivores and eat a variety of flying insects (Clare *et al.*, 2014), so we used the average composition of flying insects found in a typical diet for little brown bats as proportions (i.e. 71.2% protein, 18.4% fat and 8.8% carbohydrate; Kurta *et al*., 1989). To calculate whole-animal MR, we used the average mass of little brown bats captured in central Manitoba in early spring (i.e. 8.47 g; Jonasson and Willis, 2011) before converting all values of heat production to kilojoules.

In central Canada, insectivorous bats emerge from hibernation in late April (Norquay and Willis, 2014) at a time when they almost certainly must rely on torpor to reduce energy expenditure and balance their energy budgets, despite the fact that torpor will delay gestation and parturition (Lewis, 1993). To understand how roost microclimate might influence energy expenditure for reproductive females and survivors of WNS emerging in early spring (April–May), we quantified daily thermoregulatory energy expenditure under four roost-microclimate scenarios approximating natural conditions or microclimate manipulations that wildlife managers could potentially employ in the field, as follows: Scenario 1, outside *T*_a_ (i.e. assuming that *T*_roost_ = *T*_a_); Scenario 2, *T*_roost_ reflecting conditions in a natural maternity roost (Lausen and Barclay, 2006); Scenario 3, an artificially heated bat house, in which *T*_roost_ is cycled on and off to reduce thermoregulatory costs during periods when reproductive bats are typically normothermic while allowing some torpor expression; and Scenario 4, a heated bat house with *T*_roost_ consistently maintained within the thermoneutral zone (TNZ; i.e. 32°C; Studier and O'Farrell, 1976; Fenton and Barclay, 1980).

For Scenario 1, we used maximal and minimal daily *T*_a_ recorded from April to May at the meteorological station in Hodgson, Manitoba (51.11°N, 97.27°W) over a 10 year period from 1996 to 2005 (Environment Canada, 2014). This site is the closest weather station to Lake St George Caves Ecological Reserve, the largest known little brown bat hibernaculum in central Canada. We used these *T*_a_ values and Equations 1–4 to calculate hourly thermoregulatory energy expenditure during normothermia, cooling, steady-state torpor and rewarming for each day in April and May. For each day, we assumed that the *T*_a_ experienced by bats during normothermia was the daily maximal *T*_a_ recorded at Hodgson and that the *T*_a_ experienced during torpor was the minimal daily *T*_a_. We determined the approximate duration of each phase of torpor and arousal based on values from the literature. There are few data on torpor expression of little brown bats during the active season, but Dzal and Brigham (2013) found that pregnant female little brown bats in northern New York state, USA averaged 133 min per day in torpor (Dzal and Brigham, 2013), so for roost scenarios where bats were exposed to temperature below the TNZ (i.e. roost Scenarios 1–3) we assumed that torpor bouts lasted 133 min. The duration of active rewarming was calculated using the published rewarming rate for little brown bats (0.8°C min^−1^; Willis, 2008) and the difference between torpid and normothermic *T*_b_ using the following equations:

(7) $$\mathrm{When}\;\;\;T_{a}\;<\;T_{\mathrm{tor}-\min},\mathrm{minutes}\;\;\mathrm{to}\;\;\mathrm{rewarm}=\frac{T_{\mathrm{norm}}-T_{a}}{0.8}$$

(8) $$\mathrm{When}\;\;\;T_{a}\leq T_{\mathrm{tor}-\min},\mathrm{minutes}\;\mathrm{to}\;\mathrm{rewarm}=\frac{T_{\mathrm{norm}}-T_{\mathrm{tor}-\min}}{0.8}$$

This duration was then multiplied by the metabolic cost of warming. Thus, our model assumed that pregnant female little brown bats remained normothermic for the remaining time throughout the day (i.e. the time remaining after accounting for time spent torpid, warming and cooling).

For the other three scenarios, we used the same approach, but varied the *T*_roost_ inputs into the calculations. Data on natural *T*_roost_ of little brown bats during the active season are not available, but little brown and big brown bats often use the same kinds of structures. Therefore, for Scenario 2 (i.e. a typical maternity colony), we calculated the average difference between values for *T*_roost_ reported in the literature for building or rock crevice roosts of big brown bats vs. *T*_a_ recorded outside those roosts (4.3 ± 5.65°C; Lausen and Barclay, 2006). We then added this value to the maximal and minimal daily *T*_a_ recorded at Hodgson. As with Scenario 1 (i.e. roosting at *T*_a_), we assumed that bats rewarmed from torpor actively (i.e. using metabolic heat production) and could not exploit passive rewarming.

For Scenario 3, we calculated daily energy expenditure for bats roosting in a bat house that was heated most of the time, but allowed to cool for part of the night to facilitate short, energy-saving bouts of torpor similar to those observed for free-ranging bats (Dzal and Brigham, 2013). We set the daytime maximal *T*_roost_ in the heated bat house as 32°C (i.e. the lower end of the TNZ for little brown bats; Studier and O'Farrell, 1976; Fenton and Barclay, 1980) and used the daily minimal *T*_a_ from the Hodgson meteorological station as the minimal *T*_roost_. This model assumed that bats could rewarm from torpor passively as *T*_roost_ in the heated bat house was raised back to 32°C in the morning.

For Scenario 4, we held *T*_roost_ constant at 32°C within the TNZ so that energy expenditure during roosting was equal to BMR and normothermic *T*_b_ could be maintained with no additional thermoregulatory energy expenditure (Studier and O'Farrell, 1976; Fenton and Barclay, 1980).

### Statistical analyses

All analyses were conducted in R (version 2.14.1; R Development Core Team, 2011). To test whether bats were more likely to occupy artificially heated or unheated bat houses within each treatment group, we used McNemar's test with Yate's correction for continuity on a 2 × 2 contingency table, quantifying the number of paired observation days when at least one bat was present or when bats were entirely absent from the heated or unheated bat house. To test for a difference in use of the heated vs. unheated bat house between infected and control groups, we used Fisher's exact test on two additional 2 × 2 contingency tables, this time quantifying the presence and absence of at least one bat in the heated and unheated bat house. We were not able to bring the two groups of bats to the laboratory or introduce the bat houses at the same times; therefore, for the Fisher's exact test, we only used observations from 20 June to 23 July 2013, when both groups were present in the flight cages simultaneously with access to both bat houses.

To compare the energetic implications of our four roost scenarios, we first calculated the average daily energy expenditure for April and May of each year (i.e. from 1996 to 2005). Thus, we calculated one value of average daily energy expenditure for April 1996, one value for May 1996, one value for April 1997, and so on. We then used an ANOVA for each month with average daily energy expenditure as the response variable, Scenario (i.e. 1–4) as the predictor and year as the experimental unit. We used Tukey's honest significance test for *post hoc* analysis. Significance was assessed at the *P*<0.05 level, and all values are reported as the means ± SD.

## Results

**Figure 1:**
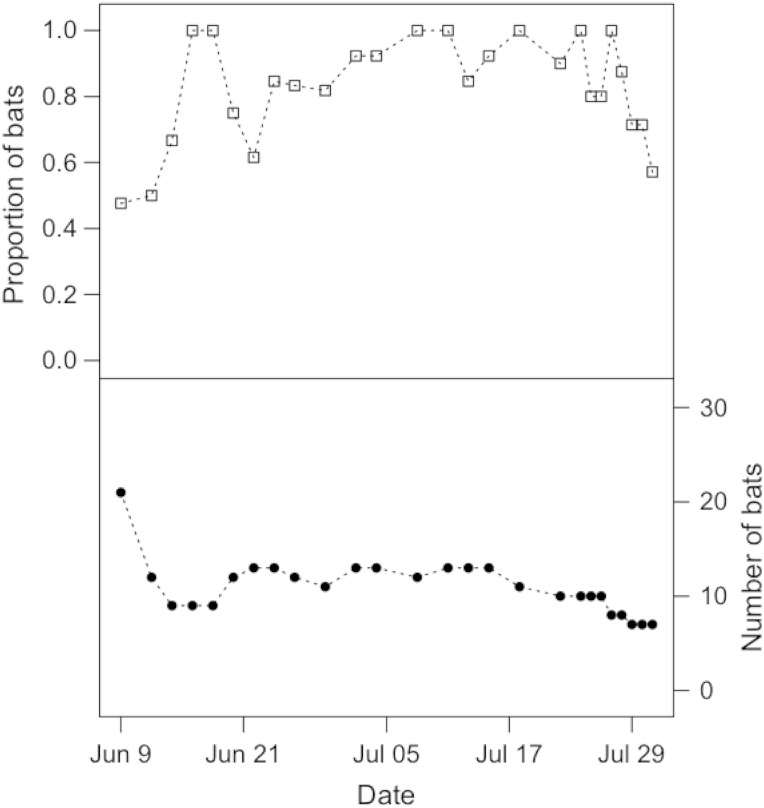
Top panel shows the proportion of control bats roosting in the heated bat house over the 2 month sampling period for healthy bats. Some bats had to be removed from the experiment over time (see Materials and methods); therefore, the bottom panel shows the number of bats remaining in the flight cage on each sampling day.

**Figure 2: COV070F2:**
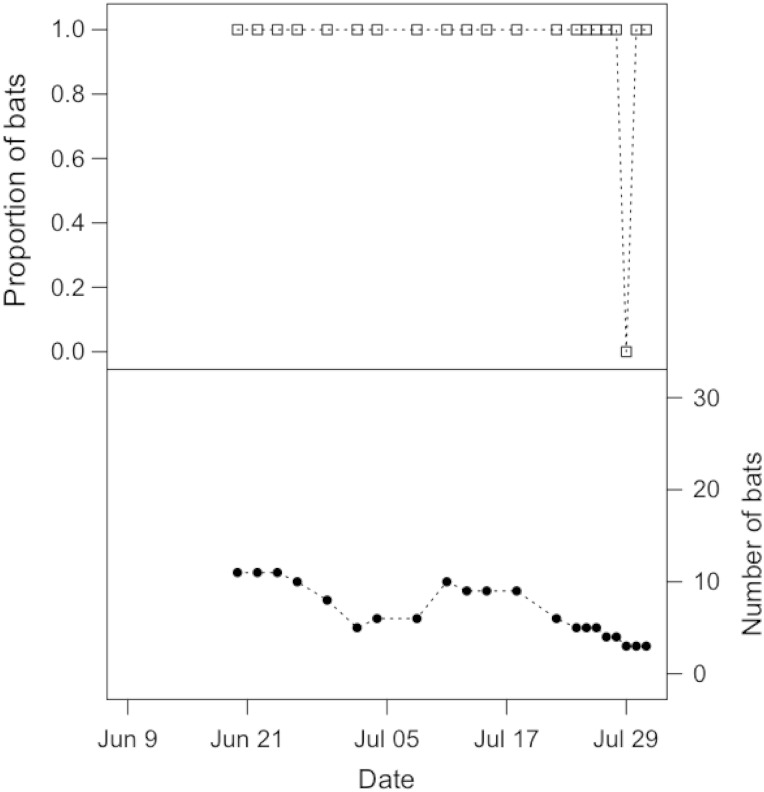
Top panel shows the proportion of infected bats roosting in the heated bat house over the 2 month sampling period. Some bats had to be removed from the experiment over time (see Materials and methods); therefore, the bottom panel shows the number of bats remaining in the flight cage on each sampling day.

**Figure 3:**
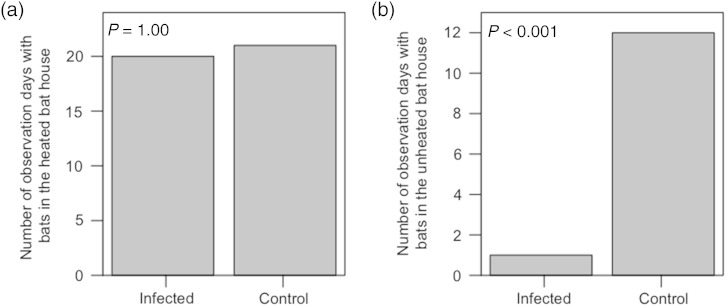
The number of observation days during which at least one bat was observed in either the heated (**a**) or unheated (**b**) bat house for bats infected with *Pseudogymnoascus destructans* and control animals. *n* = 21 observation days.

Solitary bats occasionally roosted outside of the bat houses, hanging on the aluminum mesh of the flight cages, but the vast majority of individuals roosted in the bat houses throughout their time in captivity. Control bats preferred the heated bat house (Fig. 1), and on average 82.6 ± 16.1% of individuals were observed roosting in the heated box on our observation days (Fig. 1). The number of observation days on which at least one control bat was observed in the heated bat house was greater than the number of observation days when at least one bat was observed in the unheated bat house (McNemar's $X_{1}^{2}=9.09$, *P* = 0.003; Table 2a). This preference was even stronger for infected bats, with bats almost always observed in the heated bat house (Fig. 2). On average, 95.2 ± 21.8% of bats were observed roosting in the heated box on our observation days (Fig. 2), and infected bats were significantly less likely to use the unheated bat house (McNemar's $X_{1}^{2}=15.42$, *P* < 0.001; Table 2b). At least one bat was observed in the heated bat house on an equal number of observation days for the infected and control groups (*P* = 1.0; Fig. 3a and Table 2c), but infected bats were much less likely than their healthy counterparts to use the unheated bat house (*P* < 0.001; Fig. 3b and Table 2d).[Table COV070TB1]

**Table 1: COV070TB1:** Parameter values used in the bioenergetic models to quantify thermoregulatory costs in heated and unheated roosts of little brown bats

Parameter	Value	Reference
Mass	8.47 g	Jonasson and Willis (2011)
Basal metabolic rate (BMR)	1.44 ml O_2_ g^−1^ h^−1^	Willis *et al.* (2005)
Minimal torpid metabolic rate (TMR_min_)	0.03 ml O_2_ g^−1^ h^−1^	Hock (1951), Humphries *et al*. (2002, 2005)
Normothermic temperature (*T*_norm_)	35°C	Thomas *et al.* (1990), Humphries *et al.* (2002, 2005)
Lower critical temperature (*T*_lc_)	32°C	Stones and Wiebers (1965), Humphries *et al.* (2002, 2005)
Minimal torpid temperature (*T*_tor-min_)	2°C	Hock (1951), Humphries *et al.* (2002, 2005)
Normothermic conductance (*C*_norm_)	0.2638 ml O_2_ g^−1^ °C^−1^	Stones and Wiebers (1965), Humphries *et al.* (2002, 2005)
Torpid conductance (*C*_tor_)	0.055 ml O_2_ g^−1^ °C^−1^	Hock (1951), Humphries *et al.* (2002, 2005)
*Q*_10_	1.6 + 0.26*T*_a_ − 0.006 *T*_a_^2^	Hock (1951), Humphries *et al.* (2002, 2005)
Specific heat capacity of tissue	0.131 ml O_2_ g^−1^ °C^−1^	Thomas *et al.* (1990), Humphries *et al.* (2002, 2005)

**Table 2:** Contingency tables determining whether control bats (**a**; *n* = 26) and infected bats (**b**; i.e. infected with *Pseudogymnoascus destructans*; *n* = 21) were more likely to select an artificially heated bat house or unheated bat house and whether infection status affected the presence and absence of bats in the heated bat house (**c**; *n* = 21) and unheated bat house (**d**; *n* = 21)

(a) Control bats		
	At least one bat selected the heated bat house	At least one bat did not select the heated bat house
At least one bat selected the unheated bat house	15	0
At least one bat did not select the unheated bat house	11	0
(b) Infected bats		
	At least one bat selected the heated bat house	At least one bat did not select the heated bat house
At least one bat selected the unheated bat house	0	1
At least one bat did not select the unheated bat house	20	0
(c) Presence or absence in the heated bat house		
	Control	Infected
At least one bat selected the heated bat house	21	20
At least one bat did not select the heated bat house	0	1
(d) Presence or absence in the unheated bat house		
	Control	Infected
At least one bat selected the heated bat house	12	1
At least one bat did not select the heated bat house	9	20

**Figure 4:**
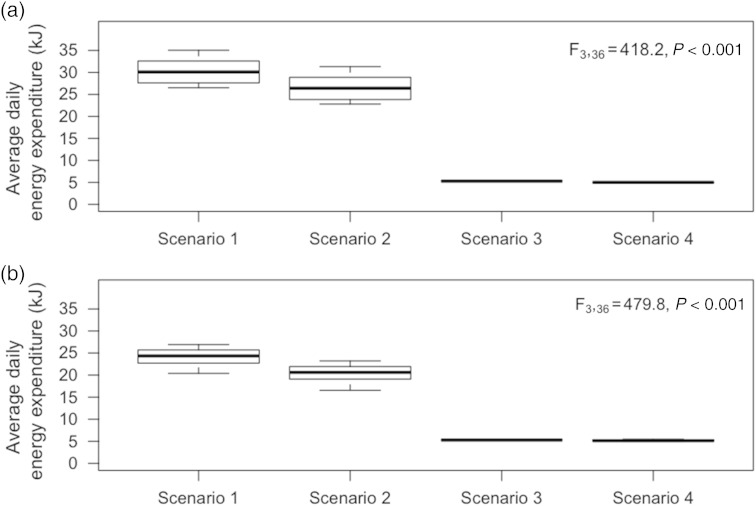
Average daily thermoregulatory energy expenditure (in kilojoules) in April (**a**) and May (**b**) for a little brown bat (*Myotis lucifugus*) roosting at ambient temperature (Scenario 1), at a temperature typical for a temperate bat maternity roost (Scenario 2), in a heated bat house with ambient temperature (*T*_a_) cycled to allow some torpor expression (*T*_a_ cycled; Scenario 3) and in a heated bat house maintained at a roost temperature within the thermoneutral zone (32°C; Scenario 4).

**Figure 5:**
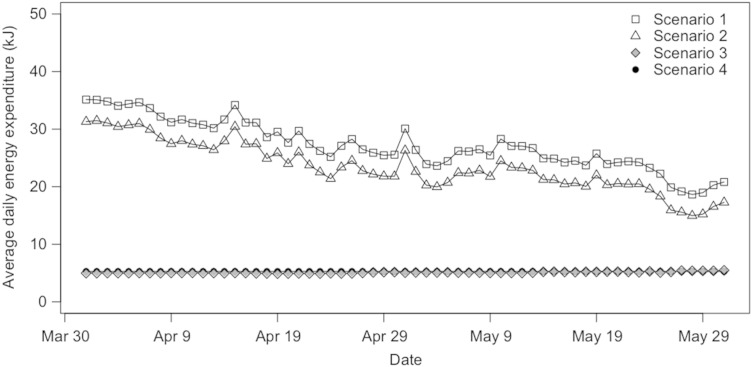
Predicted values of daily thermoregulatory energy expenditure (in kilojoules) averaged over 10 years from 1996 to 2005 during spring for a little brown bat roosting at ambient temperature (Scenario 1), at a temperature typical for a temperate bat maternity roost (Scenario 2), in a heated bat house with programmed daily fluctuation in temperature to allow some torpor expression (*T*_a_ cycled; Scenario 3) and in a heated bat house maintained within the thermoneutral zone (32°C; Scenario 4).

Based on our energetic model, there was a significant effect of roosting scenario on predicted energy expenditure during April (*F*_3,36_ = 418.2, *P* < 0.001; Fig. 4a), and all scenarios differed significantly from each other except for Scenarios 3 and 4 (Figs 4a and 5). Predicted energy expenditure was reduced by as much as 81.2% in the heated roost (Scenario 3) compared with roosting in a typical maternity colony. Likewise, during May there was also a significant effect of roosting scenario on predicted energy expenditure (*F*_3,36_ = 479.8, *P* < 0.001; Fig. 4b), and all scenarios differed significantly, except for Scenarios 3 and 4 (Figs 4b and 5). Predicted energy expenditure was as much as 74.7% lower in the heated roost (Scenario 3) compared with roosting in a typical maternity colony.

## Discussion

Our results support the potential of roosting habitat enhancement as a management tool for threatened, temperate-zone bats, in general, and as a response to WNS, in particular. We found that both infected and control bats preferred roosting in an artificially heated bat house over an unheated bat house, that bats recovering from WNS virtually always used the heated bat house, and that recovering bats were less likely than control bats to select the unheated bat house. Our model suggests that heated roosts could provide dramatic thermoregulatory energy savings during conditions typical of the post-hibernation period in central Canada, compared with energy expenditure in natural roosts. Our results also suggest the importance of protecting and possibly enhancing high-quality roosting habitat as a management action to enhance survival, reproduction and recovery of bat populations affected by WNS and other threats.

Habitat modification targeting roost or nest microclimate has shown potential as conservation tool for other taxa. For example, heated nest boxes increased reproductive fitness for birds nesting in cavity by reducing energetic costs of incubation and improving the survival of offspring (Nager and Noordwijk, 1992; Bryan and Bryant, 1999). During times of energy limitation, such as reproduction, animals should select microclimates that help to minimize energy expenditure. Although a number of studies have shown that male bats of temperate species rely heavily on torpor during summer and suggest that they should select colder roosts than females (e.g. Lausen and Barclay, 2003), data are scarce for many species. We found that male little brown bats from both infected and control groups preferentially roosted in the heated bat house (Figs 1–3a), possibly because warm *T*_roost_ could improve rates of spermatogenesis (Speakman and Thomas, 2003). To reduce impacts of our research on the wild bat population we restricted our experiment to males, but warm roost microclimates are predicted to be even more important for females. Warm microclimates reduce the energetic demands of thermoregulation during gestation and lactation and enhance the growth of offspring (e.g. Lausen and Barclay, 2003; Speakman and Thomas, 2003). However, pregnant and post-lactating bats are also known to select roosts that have cool microclimates early in the day, presumably to facilitate some use of torpor (Lausen and Barclay, 2003). For some species, the use of cool microclimates may be especially important during inclement weather, when deep torpor can slow the growth of offspring and delay parturition until conditions become more favourable (Willis *et al.*, 2006). Thus, habitat that includes a diversity of microclimates could be most beneficial for survival, reproduction and recovery from WNS. We suggest that future studies quantify *T*_roost_ preferences and their energetic implications for females of bat species facing the most pronounced impacts affected by WNS.[Fig COV070F2]

Warm roost microclimates could also enhance recovery from WNS. White-nose syndrome causes increased energy expenditure during hibernation that prematurely reduces fat stores (Warnecke *et al.*, 2012; Verant *et al.*, 2014), which means affected bats that survive are likely to emerge with minimal energy reserves in spring and must mount an energetically costly immune response while food resources are still scarce. Our results suggest that little brown bats surviving WNS may attempt to compensate for energetic costs via selection of warm roost microclimates. Warm microclimates are known to improve healing of skin lesions in other vertebrates (e.g. Anderson and Roberts, 1975) and, in our study, infected bats virtually always selected the heated bat house (Fig. 3b). It is possible that the timing of availability for each type of bat house in our experiment influenced our results. This study was one of several time-sensitive experiments running in our laboratory to address the recovery phase from WNS, and the timing of availability of bats and equipment prevented us from synchronizing the introduction of heated roosts for both groups. The infected group had a short period of initial exposure to the heated bat house before the unheated bat house was provided, whereas the control group was exposed to both heated and unheated bat houses at the same time. Therefore, infected bats had longer to acclimate to the heated bat house, meaning that the difference we observed should be interpreted cautiously. Nonetheless, free-ranging bats routinely switch between multiple roost sites in the wild (e.g. Willis and Brigham, 2004), and infected bats could have investigated both bat houses and easily switched to the unheated bat house if they preferred it. Moreover, the fact that both groups exhibited a significant preference for heated roosts supports their potential as a management tool even if infected bats do not exhibit a stronger preference in the wild.

Our models also highlight the potential benefits of warm roost microclimates for survival, reproduction and population recovery, particularly for bats with limited fat reserves. Predicted thermoregulatory energy expenditure was reduced by as much as 81.2% for bats exposed to thermoneutral *T*_roost_ with brief opportunities for torpor expression followed by passive warming, compared with maintaining normothermia in a natural roost. Although we only accounted for costs of thermoregulation in our models, our estimates are also plausible in the context of reported values of daily energy expenditure for little brown bats. Kurta *et al*. (1989) found that pregnant female little brown bats in New Hampshire and Massachusetts, USA, expended ∼29.9 kJ day^−1^ in late spring/early summer, with about 4.15 kJ h^−1^ attributed to flight. Our model, based on *T*_a_ values from a colder, more northern study site, predicted that basal plus thermoregulatory energy costs for a pregnant female little brown bat in a natural roost would amount to 23.5 kJ day^−1^ on average. If we account for foraging for approximately 2–3 h per night, this would add 8.5–13.5 kJ day^−1^ to our estimates (Kurta *et al.*, 1989), leading to a plausible range of daily energy expenditure values of 32.0–37.0 kJ day^−1^. Thus, the behavioural preference of bats for warm microclimates, combined with our estimates of predicted thermoregulatory energy expenditure, suggests the potential of artificially heated bat houses to help mitigate declines of WNS-affected bat populations.

To date, most proposed mitigations for WNS focus on treating infected bats during winter, whereas few studies have addressed the potential importance of summer habitats (Willis, 2015). Protection and enhancement of summer habitats could improve spring and summer survival and reproduction of bats that make it through the winter with WNS. As a result, they could also increase the chance that any heritable traits that provide a winter survival advantage will be passed on to offspring and accumulate in surviving populations (Q. M. R. Webber and C. K. R. Willis, unpublished data). We recommend that future studies investigate preferences for, and energetic benefits of, heated bat houses for females of temperate bat species in the wild and determine the impact of artificial heating on gestation, juvenile development and survival of bats with and without WNS. Given the large North American market for bat houses (estimated at ∼25 000 bat houses sold in the USA each year; R. Mies, Organization for Bat Conservation, personal communication), heated bat houses could theoretically be deployed on a large scale on private and public property where AC power is available. We also recommend further work to understand natural variation in *T*_roost_ experienced by WNS-affected species throughout their ranges and identify readily measurable characteristics of those roosts that predict warm *T*_roost_ so that managers can more easily identify and protect those habitats.

Despite the potential benefit of warm roost microclimates, more work is needed to address potential limitations and negative consequences of artificially heated bat houses as a management tool. For example, warm roosts that prevent bats from using torpor during spring could be counter-productive if they increase energy expenditure when food is unavailable. It is also possible that, if many bats exhibit strong preferences for heated roosts, then social network dynamics could be altered in ways that lead to enhanced pathogen or parasite transmission, including transmission of *P. destructans* (Q. M. R. Webber and C. K. R. Willis, unpublished data). Understanding the impacts of *T*_roost_ manipulation for social dynamics and pathogen or parasite transmission is important for evaluating the potential of habitat enhancement as a management strategy. Artificially eated bats houses will also be effective only for species, such as little brown bats, that regularly rely on anthropogenic structures. Other endangered bat species, such northern long-eared bats (*Myotis septentrionalis*), are unlikely to benefit because they rely more heavily on roosts in trees. Importantly, though, our results support not only artificial heating as a management tool but also the protection of the highest quality natural summer roosting habitat (i.e. forest roosts that provide the warmest roost microclimates). These kinds of habitats could be critical for helping forest-dependent species to maintain energy balance during spring recovery.

We found that both infected and control bats preferentially selected heated bat houses and that infected bats appeared to exhibit a stronger preference than control animals. The bioenergetic model we devised also highlighted the potential thermoregulatory benefits of artificially heated roosts compared with natural sites. Taken together, these results suggest that heated bat houses, in addition to protection of high-quality natural roosts with warm microclimates, could be useful as a conservation measure by improving conditions for gestation, lactation and offspring development and by enhancing recovery from WNS. Roost microclimate enhancement could be implemented by running AC or solar power to existing roosts (i.e. those often found in man-made structures or on private or public property) or designing structures that best retain solar heat and warm passively. Regardless, before this strategy is attempted on a large scale in the field, more data are needed on effects of *T*_roost_ manipulation on gestation, pup development, healing from WNS and social dynamics that could influence pathogen transmission.

## Funding

This work was supported by scholarships from the Natural Sciences and Engineering Research Council (NSERC, Canada) and University of Winnipeg Graduate Studies to A.W. and grants from the US Fish and Wildlife Service, Bat Conservation International and NSERC to C.K.R.W.
